# Refractory invasive aspergillosis controlled with posaconazole and pulmonary surgery in a patient with chronic granulomatous disease: case report

**DOI:** 10.1186/1824-7288-40-2

**Published:** 2014-01-08

**Authors:** Eda Kepenekli, Ahmet Soysal, Canan Kuzdan, Nezih Onur Ermerak, Mustafa Yüksel, Mustafa Bakır

**Affiliations:** 1 Division of Pediatric Infectious Diseases, Marmara University School of Medicine, Istanbul, Turkey; 2 Department of Chest Surgery, Marmara University School of Medicine, Istanbul, Turkey; 3 Department of Pediatrics, Division of Pediatric Infectious Diseases, Marmara University Pendik Training and Research Hospital, Mimar Sinan Street, No:41, Fevzi Cakmak Mah., Ust Kaynarca, Pendik, Istanbul, Turkey

**Keywords:** Chronic granulomatous disease, Invasive pulmonary aspergillosis, Posaconazole

## Abstract

Invasive aspergillosis is an important cause of morbidity and mortality in immunocompromised patients. Among primary immunodefiencies, chronic granulomatous disease (CGD) has the highest prevalence of invasive fungal diseases. Voriconazole is recommended for the primary treatment of invasive aspergillosis in most patients. In patients whose aspergillosis is refractory to voriconazole, therapeutic options include changing class of antifungal, for example using an amphotericin B formulation, an echinocandin, combination therapy, or further use of azoles. Posaconazole is a triazole derivative which is effective in *Aspergillosis* prophylaxis and treatment. Rarely, surgical therapy may be needed in some patients. Lesions those are contiguous with the great vessels or the pericardium, single cavitary lesion that cause hemoptysis, lesions invading the chest wall, aspergillosis that involves the skin and the bone are the indications for surgical therapy.

Chronic granulomatous disease (CGD) is an inherited immundeficiency caused by defects in the phagocyte nicotinamide adenine dinucleotidephosphate (NADPH) oxidase complex which is mainstay of killing microorganisms. CGD is characterized by recurrent life-threatening bacterial and fungal infections and by abnormally exuberant inflammatory responses leading to granuloma formation, such as granulomatous enteritis, genitourinary obstruction, and wound dehiscence. The diagnosis is made by neutrophil function testing and the genotyping.

Herein, we present a case with CGD who had invasive pulmonary aspergillosis refractory to voriconazole and liposomal amphotericine B combination therapy that was controlled with posaconazole treatment and pulmonary surgery.

## Background

Chronic granulomatous disease (CGD) is a rare inherited phagocytic disorder that results in an increased susceptibility to bacterial and fungal infections and granulomatous complications [1,2]. CGD is characterized by the inability of phagocytes (neutrophils, monocytes, macrophages) to produce reactive oxygen species due to the absence or dysfunction of the NADPH complex [2,3]. The aberrant phagocyte function characteristic of CGD increases susceptibility to infection to catalase-positive organisms (e.g., *Nocardia, Aspergillus, Serratia* etc.). Hematopoietic stem cell transplantation is the only known cure for CGD [4,5]. Antimicrobial prophylaxis with trimethoprim-sulfamethoxazole and itraconazole reduces infectious complications. Supportive treatment with interferon gamma results in better outcomes in a subgroup of variant X linked CGD patients but its routinely usage in CGD patients remains controversial [2,6].

*Aspergillus* species are ubiquitous in nature, and inhalation of infectious conidia is a frequent event. Tissue invasion is uncommon and occurs in the setting of immunosuppression. Invasive aspergillosis is an important cause of morbidity and mortality in immunocompromised patients [1,7-10]. Among primary immunodefiencies, CGD has the highest prevalence of invasive fungal diseases estimated between 20% and 40% per patient throughout their entire life [11,12]. Invasive fungal disease occurs mostly during the first 2 decades, but neonatal cases have also been reported [13]. The most common fungal genus involved is *Aspergillus* and lungs are the most frequent localization. The diagnosis is made by fungal culture of tissues or body fluids, pathological examination of affected organs, radiological findings consistent with fungal lesions (e.g., halo sign and air-crescent sign), fungal antigen assays (galactomannan and beta-1,3-D-glucan) [14]. Therapeutic options include azole derivatives, echinocandins and amphotericin B, but voriconazole is recommended as the first-line therapy [14]. Although diagnostic and therapeutic interventions improved in last decades many patients usually requires salvage therapy [14]. In patients whose aspergillosis is refractory to voriconazole, therapeutic options include a change of class using an amphotericin B formulation or an echinocandin, further use of azoles. Salvage treatment with posaconazole have been shown to be safe and effective [15,16].

Herein, we present a case with CGD who had invasive pulmonary aspergillosis refractory to voriconazole and lipozomal amphotericine B combination therapy that was controlled with posaconazole treatment and pulmonary surgery.

## Case

**Figure 1:**
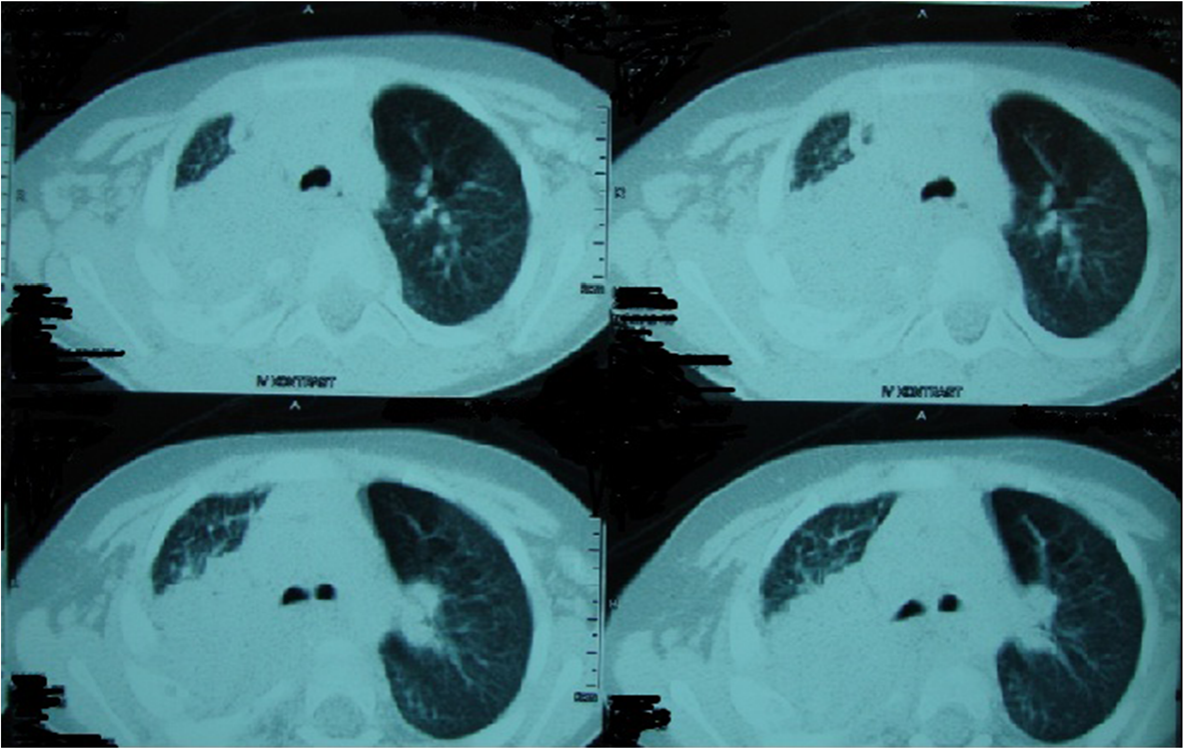
**Computerized tomography findings consistent with expansive pulmonary involvement**.

**Figure 2:**
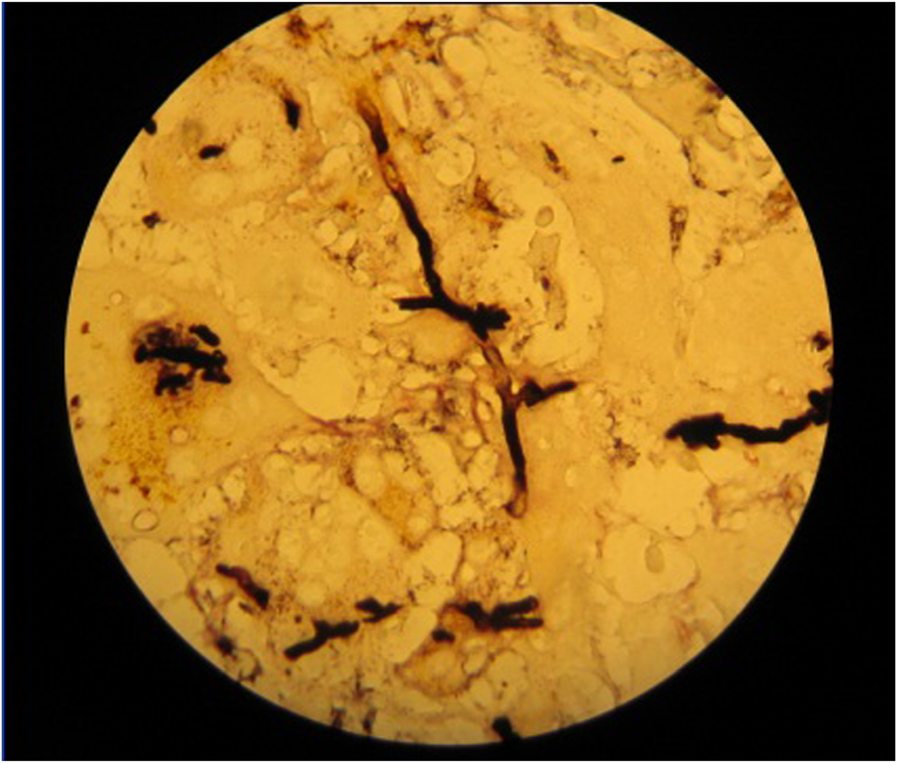
**Vertical fungal hyphaes in PAS and silver staining of the lung resection specimens**.

**Figure 3:**
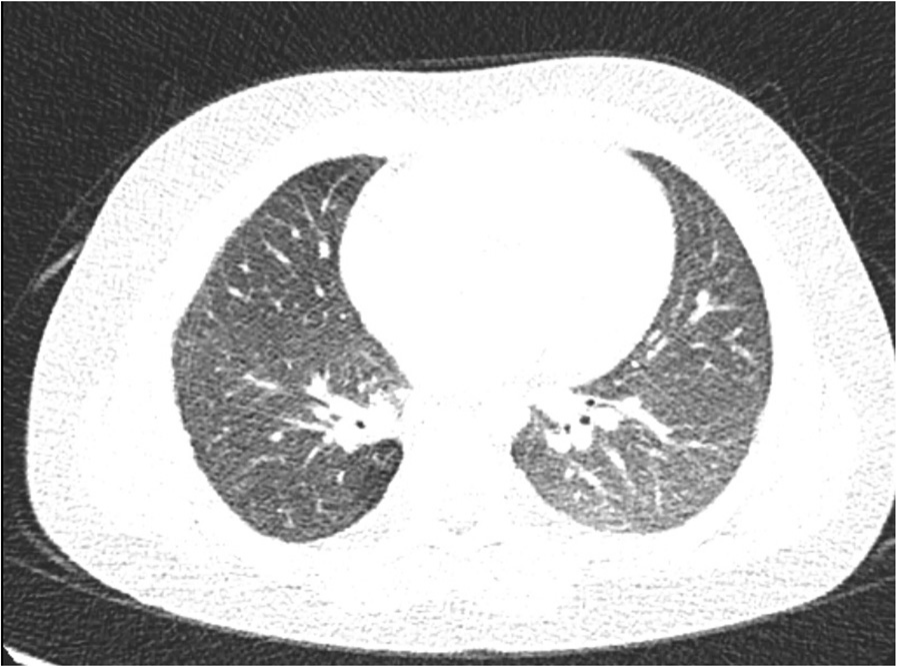
**Thorax computerized tomography imaging which is not containing fungal lesions after surgery and posaconazole theraphy**.

A 30 month-old boy presented with fever, cough, swelling and purulent discharge on upper part of his back consistent with abscess formation. Fungal culture from this abscess yielded *Aspergillus* spp. that was susceptible to voriconazole and amphotericin B but resistant to caspofungin. Voriconazole was given as initial monotherapy and then combined with liposomal amphotericin B because of progression of the lesions detected on computerized tomography (CT). Diagnosis of CGD was suggested by the presence of CGD history in the mother’s family and an extraordinary pathogen existence in the fungal culture of the abcess. The diagnosis was confirmed with the nitroblue tetrazolium and dihidro-rhodamin oxidase assay. He was defined as X-linked CGD and gp91 CYBB gene, exon7 c.742dupA,p.Ile248AsnfsX36 molecule was defective. Interferon gamma and trimethoprim-sulfamethoxasole were given for supportive care and antimicrobial prophylaxis. Constriction of the right bronchie, consolidation of the right upper lobe including air broncograms and fistulization to the skin was detected in thorax CT (Figure 1). Antifungal combination therapy with voriconazole and amphotericin B was given for six months but the lesions did not regress and caused costal bone destruction. He underwent chest surgery and his destroyed right upper pulmonary lobe, upper segment of the lower lobe, two necrotic costal bones and the tract of the fistula were resected. Vertical fungal hyphaes, supurative granulomatous inflamation, fibrosis, destruction of the bone trabeculas in PAS and silver staining of surgical resection specimens were observed in pathological examination (Figure 2). Antifungal treatment was continued with posaconazole monotherapy right after surgery. Hepatic transaminase levels, serum electrolyte levels, erythrocyte sedimentation rate, C-reactive protein, complete blood count, electrocardiography (ECG) monitorized in monthly period and all were normal. Serum galactomannan antigen was 2,09 and positive right before surgery, then it was negative repeatedly. Thorax CT was repeated in every six months. In the thirty-six months follow up since from pulmonary surgery to date, no any complaints or fungal pulmonary or bone lesions were observed and posaconazole therapy was given continuously (Figure 3).

## Conclusions

Galactomannan, a major constituent of *Aspergillus* cell walls that is released during the growth of hyphae, can usually be detected by enzyme immunoassay in serum, bronchoalveolar lavage fluid, cerebrospinal fluid, and pleural fluid [14]. But galactomannan is quite insensitive, even in the setting of proven infection in CGD [12]. In our patient galactomannan antigen was positive only for once before surgery and posaconazole therapy, then it was negative repeatedly.

Voriconazole is the primary therapy option for invasive pulmonary aspergillosis. Alternative therapies are liposomal and lipid complex amphotericin B, echinocandins and other azole derivatives such as posaconazole and itraconazole. The oral bioavailability of the azole derivatives is an advantage for the maintenance therapy after initial therapy [14]. Posaconazole is an extended spectrum triazole with invivo and invitro activity against *Aspergillus* species but dosage in pediatric patients has not been defined [14-16]. The posaconazole releated side effects are nausea, vomiting, anorexia, abdominal pain, dehidratation, fever, rash, throat and chest pain, headache, blurred vision and sleeplessness. The patient should be monitored for liver transaminase elevation, hypocalcemia, hypomagnesemia, hypokalemia, hyperglycemia, pancytopenia, hypertension, hypotension and, cardiac rhythm abnormalities such as QT interval prolongation [14-16]. Posaconazole was given 200 mg 4 times a day with meals to our patient right after the surgery. He received posaconazole for 36 months without any side effects.

Surgery may be useful in lesions that are contiguous with the great vessels or the pericardium, single cavitary lesion that cause hemoptysis, lesions invading the chest wall or in aspergillosis that involves the skin and the bone. Surgical resection of devitalized bone and cartilage is important for curative intent [14]. In our patient, destroyed right upper pulmonary lobe, upper segment of the right lower lobe, two necrotic costal bones and the tract of the fistula were resected, when disease progression was observed during the combined antifungal therapy.

In conclusion, we think, resection of the nonfunctional pulmonary parenchyma and devitalized bone and cartilages increased the success of posaconazole maintenance therapy.

## Consent

Written informed consent was obtained from the patient’s parent for the publication of this report and any accompanying images.

## Competing interests

The authors declare that they have no competing interests.

## Authors’ contributions

MY and NOE carried out the surgical intervention. EK wrote the manuscript. CK, AS and MB contributed to the writing of the manuscript and managed the medical therapy. All authors read and approved the final manuscript.
